# Frontal plane movement of the pelvis and thorax during dynamic activities in individuals with and without anterior cruciate ligament injury

**DOI:** 10.1016/j.knee.2018.06.002

**Published:** 2018-12

**Authors:** Jennifer L. Davies, Kate Button, Valerie Sparkes, Robert W. van Deursen

**Affiliations:** School of Healthcare Sciences, Cardiff University, Eastgate House, 35–43 Newport Road, Cardiff CF24 0AB, UK; Arthritis Research UK Biomechanics and Bioengineering Centre, Cardiff University, The Sir Martins Evans Building, Museum Avenue, Cardiff CF10 3AX, UK

**Keywords:** Trunk, Single-leg, Rehabilitation, ACL

## Abstract

**Background:**

For elite athletes with anterior cruciate ligament (ACL) reconstruction, reducing pelvis and trunk obliquities is a common goal of rehabilitation. It is not known if this is also a suitable goal for the general population. This study aimed to quantify pelvis and thorax obliquities during dynamic activities in individuals from the general population with and without history of ACL injury.

**Methods:**

Retrospective analysis of cross-sectional data from 30 participants with ACL reconstruction, 28 participants with ACL deficiency (ACLD), and 32 controls who performed overground walking and jogging, single-leg squat, and single-leg hop for distance. Pelvis and thorax obliquities were quantified in each activity and compared across groups using one-way ANOVA. Coordination was quantified using cross covariance.

**Results:**

In the stance phase of walking and jogging, pelvis and thorax obliquities were within ±10° of neutral and there was a negative correlation between the two segments at close to zero phase lag. In single-leg squat and hop, range of obliquities varied across individuals and there was no consistent pattern of coordination. Eight ACLD participants felt unable to perform the single-leg hop. In the remaining participants, range of pelvis (p = 0.04) and thorax (p = 0.02) obliquities was smaller in ACLD than controls.

**Conclusions:**

In challenging single-leg activities, minimal frontal plane motion was not the typical movement pattern observed in the general population. Coordination between the pelvis and thorax was inconsistent within and across individuals. Care should be taken when considering minimising pelvis and thorax obliquities in patients with ACL injury.

## 1 Introduction

Rehabilitation is recommended for individuals with anterior cruciate ligament (ACL) rupture who have surgical reconstruction (ACLR) and individuals who manage their injury conservatively and remain ACL deficient (ACLD). A core aspect of rehabilitation is progressive limb loading and the performance of dynamic activities [1], [2]. These activities require coordination of the whole body, and indirect evidence suggests that control of the trunk may influence ACL injury risk [3], possibly through an effect on external knee abduction moment [4], [5], [6].

Trunk and pelvis control is linked to lower extremity valgus [7], [8], and is one aspect of rehabilitation of lower-limb injuries [7], [9], [10]. Indeed, for elite athletes with ACLR, the absence of pelvis and trunk movement in the frontal plane during single-leg movements is one marker of progression through rehabilitation [11]. However, there is little information on the movement of the pelvis and trunk segments in the frontal plane after ACL injury in non-elite athletes, and no guidelines for physiotherapists treating this population. It is therefore not clear what amount of frontal plane motion is expected, and what is a realistic and appropriate goal for rehabilitation after ACL injury in the general population.

Coordination between the pelvis and thorax in the frontal plane during dynamic movements can be categorised as en bloc, where the two segments tilt in the same direction, or articulated, where the two segments tilt in opposite directions. Coordination during walking develops from an en bloc strategy in toddlers learning to walk, to an articulated operation of the hip–shoulder unit [12]. The type of pelvis and trunk coordination present in whole-body, dynamic activities that are commonly used in rehabilitation after ACL injury is not known.

The aim of this study was to quantify pelvis and trunk movement and coordination in the frontal plane during dynamic activities in individuals from the general population with and without a history of ACL injury. This was achieved by retrospective analysis of existing data, parts of which have previously been published [13], [14], [15]. Previous analyses of these data have revealed that, despite having completed physiotherapy, the participants with ACL injury had incomplete recovery [13], [14], [15]. We hypothesised that this would be reflected in suboptimal pelvis and trunk control in the frontal plane, and thus hypothesised that individuals with a history of ACL injury would have greater range of pelvis and trunk movement in the frontal plane than individuals without a history of ACL injury.

## 2 Materials and methods

This was a retrospective analysis of cross-sectional data collected as part of a previous study. Approval for the study was obtained from the Research Ethics Committee for Wales (10/MRE09/28).

### 2.1 Participants

Participants with ACL injury were recruited from five physiotherapy departments within the Cardiff and Vale University Health Board (Cardiff, UK). Participants without lower-limb injury were recruited from the Cardiff region. Inclusion criteria were age 18–50 years; no neurological disorder that affects lower-limb mobility; no pathology or injury (other than ACL rupture with or without accompanying meniscal tear or collateral ligament sprain but without accompanying posterior cruciate ligament injury for ACLR and ACLD participants) that affects lower-limb mobility; and no previous trauma to the lower-limb that required clinical intervention. All ACLR participants had a four-strand single-bundle gracilis–semitendinosus tendon graft reconstruction with an anatomical tunnel position. All ACLR and ACLD participants had completed rehabilitation. As this was a retrospective analysis of existing data, no a priori power calculation was performed for this analysis.

Knee function was evaluated using the International Knee Documentation Committee Subjective Knee Form [16]. Fear of re-injury was evaluated using the Tampa Scale of Kinesiophobia (TSK) [17] modified to specifically evaluate fear of knee re-injury [18]. Sports activity level (general activity level for control participants, post-injury activity level for ACLD and ACLR participants) was measured using the Cincinnati Sports and Activity Scale [19].

### 2.2 Protocol

Data were analysed from four activities: overground walking at a self-selected speed, overground jogging at a self-selected speed, single-leg squat, and single-leg hop for distance. The order of activities was not randomised, as it was considered that it may have been unsafe to start with one of the more challenging activities. Activities were performed in the order listed above, and participants only progressed if they did not have symptoms and it was judged by a physiotherapist to be safe to continue. Participants were given rest time between activities to prevent fatigue. All activities were performed while the participant was wearing their own footwear. Participants were allowed to practice each task to familiarise themselves with the testing procedures. For walking and jogging trials, the participant travelled across a 10-m walkway in the laboratory over force plates (Kistler Instruments Ltd., Switzerland) embedded in the floor. Participants were not made aware of the force plates and were not instructed to step onto the force plates. Trials were only accepted if there was a clean foot strike on the force plate, and data were analysed for the stride that contacted the force plate. Data were only analysed for the stance phase of the stride. For single-leg hop-for-distance trials, participants were instructed to hop as far as they felt possible while maintaining a stable landing. The starting position was adjusted so that the participant landed on the force plate. Trials were only accepted if stability was maintained on landing, with no visible onward hop or foot motion. Walking, jogging, and single-leg hop-for-distance trials were repeated until five successful trials had been recorded. For single-leg squat trials, participants performed up to 10 repetitions of the movement to a self-selected depth. For further details please see [13], [14], [15].

### 2.3 Data collection

Data collection took place between November 2010 and November 2012. Retroreflective markers were placed according to the Plug-in-Gait marker set (Vicon Motion Systems Ltd., Oxford, UK). The same experimenter placed markers for all subjects, and marker placements were verified by a second experimenter who was a musculoskeletal physiotherapist with expertise in palpation of anatomical landmarks. The position of these markers was recorded at 250 Hz using an eight-camera motion analysis system (Vicon 512, Vicon Motion Systems Ltd.). Force plate data (Kistler Instruments Ltd.) were collected at 1000 Hz.

### 2.4 Data analysis

All analyses were performed using a custom-written code (Matlab 2015a; The Mathworks Inc., Natick, MA). The vertical axis of the trunk was defined as the line from T10 to C7, meaning that this segment incorporates only the upper part of the trunk. This was performed to separate it from the pelvis, enabling quantification of two distinct segments. To avoid confusion, this will be referred to as the thorax segment. The angle of the pelvis and thorax segments was expressed relative to a reference system in which z is vertical in the fixed laboratory reference system, and x and y rotate about the vertical axis with the segment of interest. This eliminates the need for the body motion to be perfectly aligned with the axes of the fixed laboratory reference system, and enables segment angles to be interpreted in motions performed out of line with the fixed laboratory reference system [20].

obliquities was defined as the angle of rotation of the segment about the anterior–posterior axis of the reference system. Euler angles were calculated using the Matlab function rotro2eu (part of the Voicebox Toolbox, Department of Electrical Engineering, Imperial College London) using the sequence rotation, obliquities, tilt, as described by Baker [21]. obliquities was expressed relative to the side performing the activity (side of the injured leg for ACLD and ACLR participants and of the dominant leg for control participants). The dominant leg was defined as the limb with which participants would feel most comfortable performing a single-leg hop. Positive values indicate a rotation of the segment up on the side performing the activity.

Data were quantified over pre-defined analysis windows (Table 1). In each analysis window, pelvis and thorax obliquities were quantified using the minimum and maximum angle and the range of motion. The coupling between the two segments was quantified using cross covariance, calculated using the xcov function in Matlab.

**Table 1:** Definition of analysis windows.

Activity	Window for analysis	Start	End
Walking	Stance phase of the selected leg^a^	Vertical GRF > 5 N	Vertical GRF < 5 N
Jogging	Stance phase of the selected leg^a^	Vertical GRF > 5 N	Vertical GRF < 5 N
Single-leg squat	Entire movement	Start of knee flexion	End of knee flexion
Single-leg hop for distance	Landing phase	Vertical GRF > 5 N	Peak knee extension after maximum flexion

a The selected leg was the injured leg for anterior cruciate ligament reconstructed and deficient participants and the dominant leg for control participants. GRF, ground reaction force.

### 2.5 Statistical analysis

The distribution of data for each outcome measure was visually inspected using Q–Q plots. For each activity, minimum, maximum and range of pelvis and thorax obliquities were compared across groups (control, ACLR, ACLD) using a one-way analysis of variance. Significant effects (p < 0.05) were investigated using Bonferroni-corrected t-tests. Due to large inter-individual variability in cross-covariance functions for some activities, it was not possible to identify a single peak that was representative of the function for all participants. Cross covariance functions were therefore not compared statistically, but are presented for qualitative interpretation.

## 3 Results

Data were processed from 60 individuals with ACL injury (n = 30 ACLR; n = 30 ACLD) and 35 controls. Five participants (n = 2 ACLD and n = 3 controls) were excluded due to poor data quality. The characteristics of the remaining 90 participants are shown in Table 2. All participants performed walking, jogging, and single-leg squat trials. All control and ACLR participants performed the single-leg hop for distance trials, but only 20 of 28 ACLD participants performed this activity (further details in Section 3.3).

**Table 2:** Participant characteristics.

	Control (N = 32)		ACLR (N = 30)		ACLD (N = 28)		p
Age (years)	27.5 (6.8)	[32]	30.5 (9.7)	[30]	31.0 (7.8)	[28]	0.196
Gender	10 F; 22 M	[32]	9 F; 21 M	[30]	5 F; 23 M	[28]	–
Height (m)	1.74 (0.11)	[31]	1.74 (0.07)	[29]	1.78 (0.09)	[28]	0.247
Mass (kg)	75.3 (17.3)	[31]	80.0 (10.0)	[29]	81.9 (14.8)	[28]	0.197
Body mass index (kg/m^2^)	24.6 (3.6)	[31]	26.5 (3.6)	[29]	25.9 (4.0)	[28]	0.144
Time since injury (months)	–		23 (15)	[25]	19 (10)	[19]	0.888
Time since surgery (months)	–		13 (8)	[25]	–		–
IKDC subjective knee form score	–		82 (14)	[29]	62 (12)	[25]	0.773
Tampa Scale of Kinesiophobia score	–		24 (5)	[29]	31 (5)	[28]	0.907
Cincinnati Sports and Activity Scale score	83 (19)	[31]	86 (15)	[30]	73 (18)*	[28]	0.001

Data are mean (standard deviation) [n]. p values are from one-way analysis of variance when there are three groups, and independent t-test when there are two groups. For some variables, data were not available for all participants. The number of participants included for each variable is shown in square brackets. ACLD, anterior cruciate ligament deficient; ACLR, anterior cruciate ligament reconstructed; IKDC, International Knee Documentation Committee. Asterisk indicates non-corrected p value from post-hoc pairwise comparisons was < 0.05.

**Figure 1:**
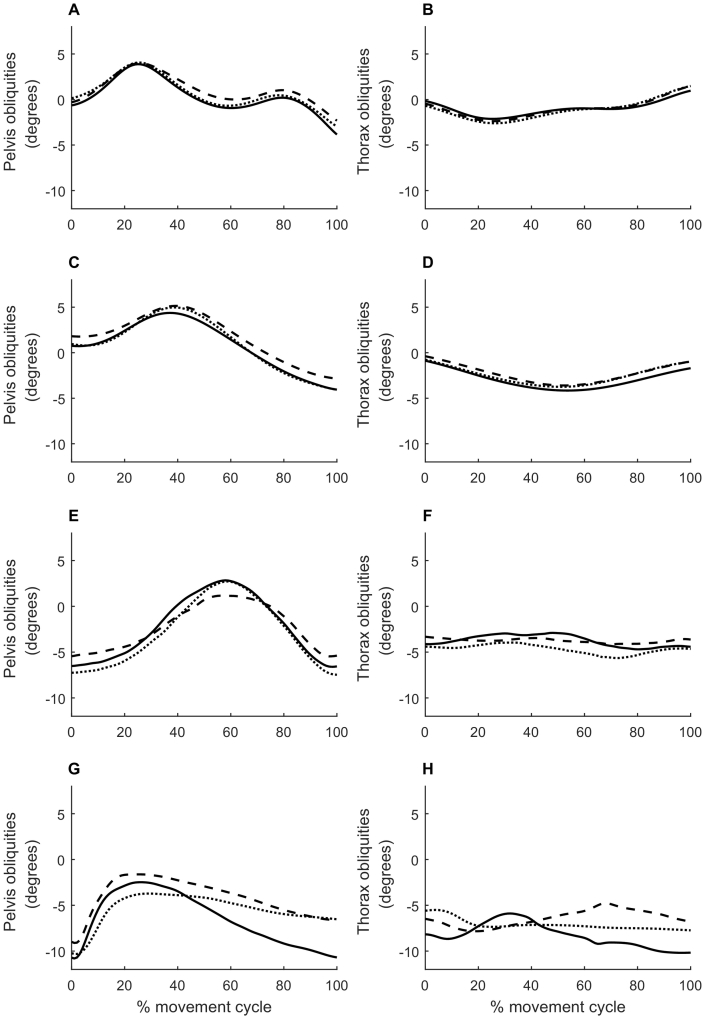
Time course of pelvis and thorax obliquities. Pelvis (left column) and thorax (right column) obliquities in the stance phase of walking (A, B), the stance phase of jogging (C, D), a single-leg squat (E, F), and the landing phase of a single-leg hop for distance (G, H). Data are the mean across all participants with no history of anterior cruciate ligament (ACL) injury (controls; solid line) and all participants with a history of ACL rupture who had undergone surgical reconstruction (ACLR; dashed line) or remained ACL deficient (ACLD; dotted line). Positive values indicate a rotation of the pelvis segment up on the side performing the activity, or a lean of the thorax away from the side performing the activity.

**Figure 2:**
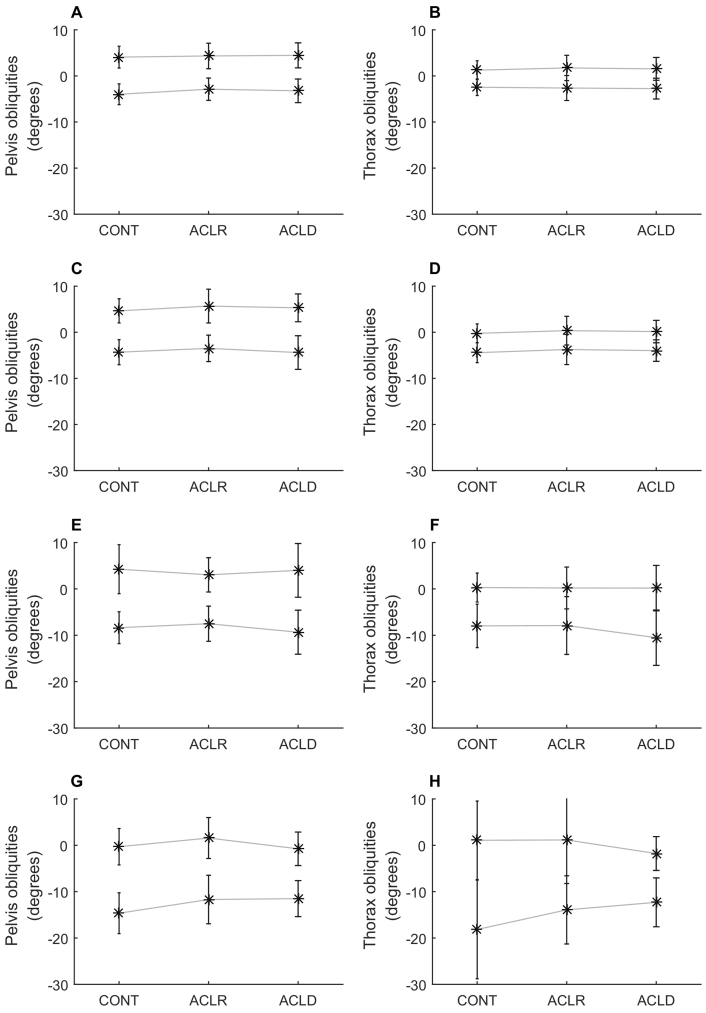
Summary of pelvis and thorax obliquities. Minimum and maximum pelvis (left column) and thorax (right column) obliquities in the stance phase of walking (A, B), the stance phase of jogging (C, D), a single-leg squat (E, F), and the landing phase of a single-leg hop for distance (G, H). Data are the mean across all participants with no history of anterior cruciate ligament (ACL) injury (controls; CONT) and all participants with a history of ACL rupture who had undergone surgical reconstruction (ACLR) or remained ACL deficient (ACLD). Error bars indicate standard deviation. Positive values indicate a rotation of the pelvis segment up on the side performing the activity, or a lean of the thorax away from the side performing the activity. There was a significant effect of group on minimum pelvis obliquities (p = 0.038), range of pelvis obliquities (p = 0.035) and range of thorax obliquities (p = 0.016) in the single-leg hop for distance.

**Figure 3:**
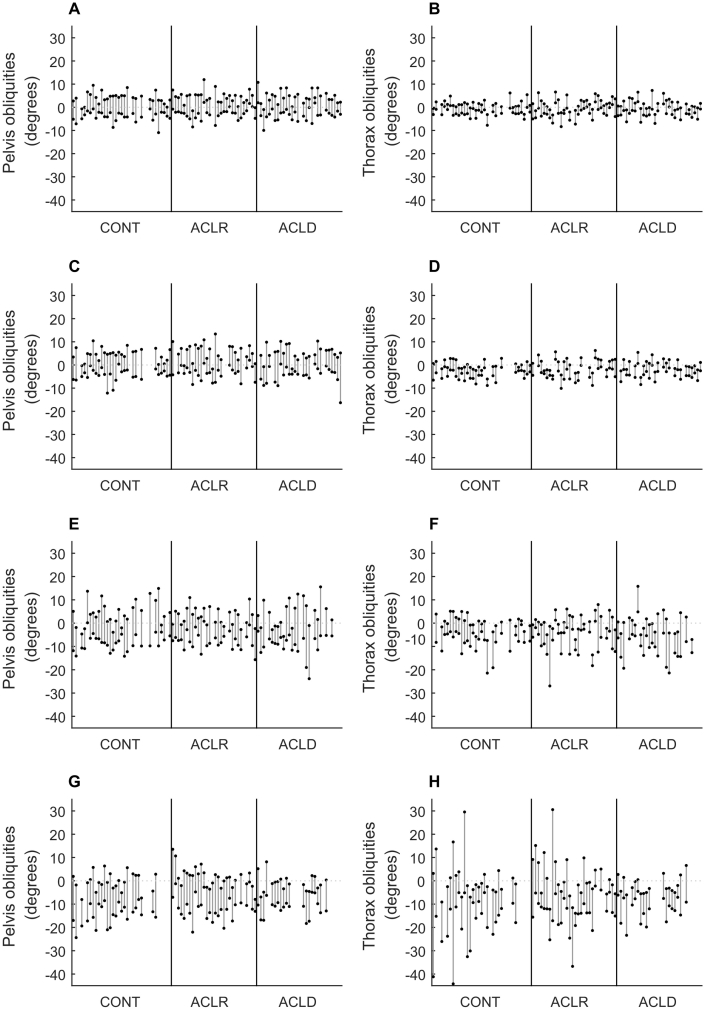
Range of pelvis and thorax obliquities for individual participants. Minimum and maximum pelvis (left column) and thorax (right column) obliquities in the stance phase of walking (A, B), the stance phase of jogging (C, D), a single-leg squat (E, F), and the landing phase of a single-leg hop for distance (G, H) for each participant. Data are shown for participants with no history of anterior cruciate ligament (ACL) injury (controls; CONT) and participants with a history of ACL rupture who had undergone surgical reconstruction (ACLR) or remained ACL deficient (ACLD). Vertical black lines indicate the boundaries of each group. Positive values indicate a rotation of the pelvis segment up on the side performing the activity, or a lean of the thorax away from the side performing the activity.

**Figure 4:**
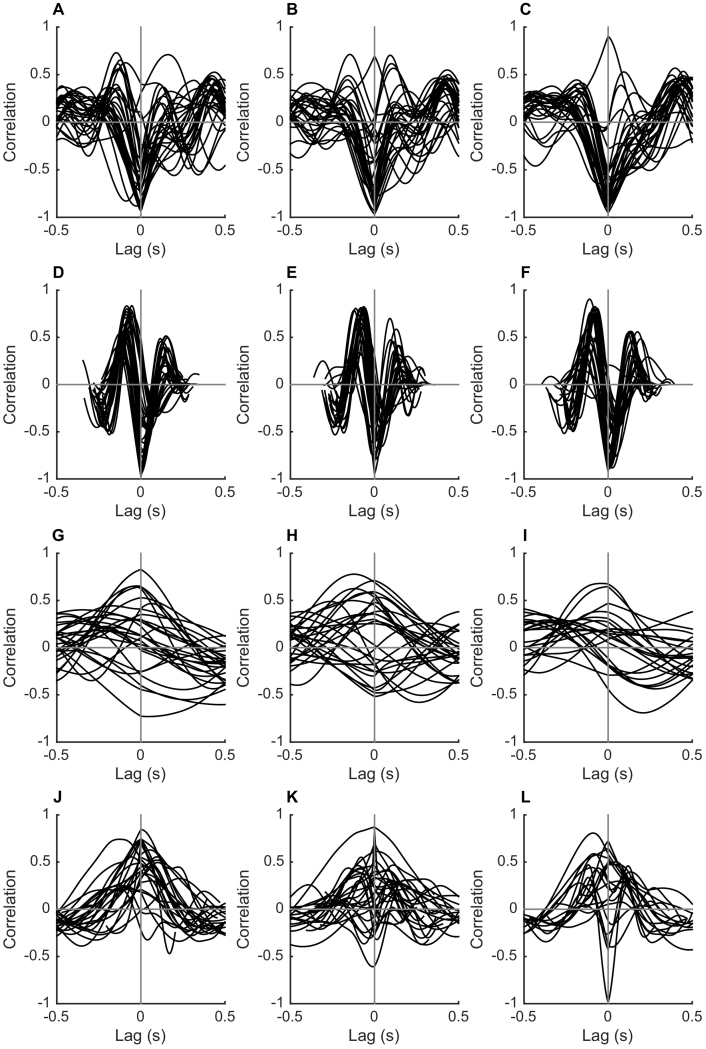
Cross covariance function between pelvis and thorax obliquities. The cross covariance function between pelvis and thorax obliquities in the stance phase of walking (top row; A–C), the stance phase of jogging (second row; D–F), a single-leg squat (third row; G–I), and the landing phase of a single-leg hop for distance (bottom row; J–L). Data are shown for each participant with no history of anterior cruciate ligament (ACL) injury (controls; left column) and each participant with a history of ACL rupture who had undergone surgical reconstruction (ACLR; centre column) or remained ACL deficient (ACLD; right column). Positive cross-covariance values indicate that the two segments followed the same movement pattern, i.e., en bloc control, and negative values indicate that they followed an opposing movement pattern, i.e., counter-movement control. Cross covariance is shown at different time shifts (lags) between the two signals (pelvis and thorax obliquities). Negative lag values on the abscissa indicate that the thorax lagged the pelvis; positive values indicate that the thorax led the pelvis. For example, a correlation of one at a time lag of zero would indicate that the segments moved perfectly in unison. A correlation of one at a time lag of five milliseconds would indicate that the pattern of movement was identical in the pelvis and thorax, but the thorax tilted five milliseconds before the pelvis.

Pelvis and thorax obliquities are shown for each activity in Figure 1. Minimum and maximum pelvis and thorax obliquities throughout each activity are shown in Figure 2 (group averages) and Figure 3 (individual participants). The cross-covariance function between pelvis and thorax obliquities for each participant for each activity is shown in Figure 4.

### 3.1 Walking and jogging

In the stance phase of walking and jogging, the pelvis and thorax were in neutral obliquities at heel strike. The pelvis then tilted so that the stance side was higher than the swing side (positive obliquities) in mid-stance, before tilting in the opposite direction just prior to toe-off (Figure 1A and C). The thorax tilted in the opposite direction (Figure 1B and D), resulting in counter-movement control of the two segments. There was no significant effect of group on minimum, maximum or range of pelvis or thorax obliquities (p = 0.136–0.962; Figure 2A–D). The range of pelvis obliquities varied from two to 17° across participants in walking and from two to 21° in jogging (Figure 3A and C, respectively). The range of thorax obliquities varied from one to eight degrees across participants in walking and jogging (Figure 3B and D, respectively).

In the majority of participants there was a strong negative correlation between pelvis and thorax obliquities at close to zero phase lag (Figure 4A–F). This supports the presence of counter-movement control of the two segments, as seen in the time series (Figure 1A–D).

### 3.2 Single-leg squat

At the start of the single-leg squat, the pelvis was tilted down (negative obliquities) on the squatting side, that is, the weight-bearing limb. As the movement progressed this tilt reduced, and the pelvis was close to neutral or tilted up on the squatting side at the bottom of the squat, before returning to the starting position at the end of the squat (Figure 1E). The group average time series show little movement of the thorax across the squat (Figure 1F). There was no significant effect of group on minimum, maximum or range of pelvis or thorax obliquities (p = 0.143–0.994; Figure 2E–F). Post hoc analysis indicated that there was a significant effect of group on maximum knee flexion in the single-leg squat (p = 0.019), whereby the control group had significantly greater knee flexion (78 [11]°) than the ACLR (70 [18]°; p = 0.131) and ACLD (68 [7]°; p = 0.002) groups, with no difference between ACLR and ACLD groups. However, including maximum knee flexion as a covariate in the analysis did not change the results, and there remained no significant effect of group on minimum, maximum or range of pelvis or thorax obliquities (analysis of covariance p = 0.156–0.987).

**Figure 5:**
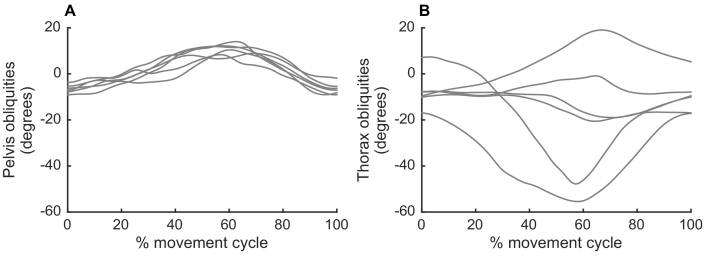
Example time course of pelvis and thorax obliquities for a single participant. Pelvis (left column) and thorax (right column) obliquities in each of five single-leg squat trials performed by a participant with a history of ACL rupture who had undergone surgical reconstruction. Positive values indicate a rotation of the pelvis segment up on the side performing the activity, or a lean of the thorax away from the side performing the activity.

There was substantial inter-individual variation in pelvis and thorax obliquities during the single-leg squat, and the range of motion for each participant is shown in Figure 3. The range of pelvis obliquities varied from three to 27° across participants, and the range of thorax obliquities varied from two to 26° (Figure 3E–F). This range of thorax movement is not reflected in the group averaged time series (Figure 1F). Closer inspection of the data revealed that this is because of a lack of consistent pattern of thorax obliquities across and within participants. This is demonstrated in Figure 5, which shows five single-leg squats from a single participant. Although pelvis obliquities showed a consistent pattern across trials, thorax obliquities showed no consistent pattern. These within-individual variations were lost by the process of averaging across trials. Similarly, between-individual variations were lost by the process of averaging across participants, and the group ensemble averages therefore showed little variation. This absence of consistent coordination between the pelvis and thorax is reflected in the cross covariance functions, which exhibit no consistent pattern (Figure 4G–I).

### 3.3 Single-leg hop for distance

Eight of the 28 ACLD participants felt unable to perform a maximal single-leg hop for distance, and did not perform these trials. Compared to the 20 ACLD participants who performed the single-leg hop for distance, these eight participants had greater fear of reinjury (mean [standard deviation] TSK score 34.9 [3.2] vs. 29.7 [5.5]; p = 0.020) and a tendency for poorer knee function (International Knee Documentation Committee Subjective Knee Form score 55.7 [8.5] vs. 65.5 [11.5]; p = 0.053) and lower sports activity level (Cincinnati Sports and Activity Scale score 63.1 [23.1] vs. 76.5 [15.1]; p = 0.082). All control and ACLR participants performed the single-leg hop for distance.

At the start of the landing phase of the single-leg hop for distance, the pelvis was tilted down (negative obliquities) on the hopping side. This tilt quickly reduced, and then re-appeared as the landing phase progressed (Figure 1G). The group averages show little movement of the thorax across the landing phase (Figure 1H).

There was a significant effect of group on minimum pelvis obliquities (p = 0.038; Figure 2); however, Bonferroni-corrected post-hoc comparisons revealed no significant pairwise differences. There was a significant effect of group on range of pelvis and thorax obliquities (p = 0.035 and 0.016, respectively), and post hoc tests indicated that the ACLD group had significantly less range of motion than the control group (mean [standard deviation] range of motion, 11 [three]° vs 14 [five]°, p = 0.025 for pelvis, and 11 [four]° vs 19 [three]°, p = 0.022 for thorax). There was no significant effect of group on maximum pelvis obliquities or minimum or maximum thorax obliquities.

The range of pelvis obliquities varied from four to 24° across participants, and the range of thorax obliquities ranged from five to 61° (Figure 3G–H). As for the single-leg squat, there is a discrepancy in the range of thorax obliquities evident in the group-averaged time-series (Figure 1H) and in the individual data (Figure 3G–H), and this is because of the lack of consistent pattern causing variations to be lost during averaging. This lack of consistent coordination between the pelvis and thorax is again reflected in the cross covariance functions, which show considerable variation across individuals (Figure 4J–L).

## 4 Discussion

In this study we provide a comprehensive description of pelvis and thorax obliquities in four dynamic activities commonly used in the rehabilitation of ACL injury. Our results show that during walking and jogging, the movement and coordination of the pelvis and thorax in the frontal plane were relatively stereotyped, displaying a counter-movement pattern of coordination with movement within ± 10° of neutral. By contrast, in single-leg squat and hop for distance, the range of motion varied across individuals and there was no consistent pattern of coordination. Pelvis and thorax obliquities were largely similar between individuals with and without ACL injury. The only difference between groups occurred in the most challenging exercise, a single-leg hop for distance, in which ACLD participants moved through a smaller range of pelvis and thorax obliquities than controls. Although statistically significant, the magnitude of this difference was small. However, our hypothesis that individuals with ACL injury would have greater range of pelvis and thorax obliquities was not supported in any of the activities performed.

The stereotyped pelvis and thorax obliquities observed throughout the stance phase of walking and jogging is reflected in the time series' and in the cross covariance functions. The strong negative correlation at close to zero phase lag observed in most individuals reflects counter-movement control of the two segments, a pattern that has been shown to emerge early in the development of walking [12], [22].

In the more challenging single-leg exercises of squat and hop there was a relatively consistent pattern of pelvis obliquities throughout the movements, but an inconsistent pattern of thorax obliquities. This inconsistency was observed across participants in all three groups, and also across trials within individual participants. The cross covariance functions reveal that some participants had negative correlation at close to zero phase lag in these activities, indicating counter-movement control of the two segments, and some had a strong positive correlation, indicating en bloc control, and there was no clear and consistent pattern of coordination. This variability may be due to the more challenging nature of these activities and the fact that they were less familiar to the participants than walking and jogging. Variability is high in the early stages of motor learning and decreases with practice [23], [24], and our results may reflect that participants were still in a learning mode for single-leg squat and hop activities. Although ACLR and ACLD participants had undergone rehabilitation, and thus may have had more prior experience of the single-leg hop than control participants, coordination was similarly variable in all three groups. It remains to be determined if a more stable pattern of coordination would emerge with practice, and if this coordination pattern would be en bloc or counter movement.

In the stance phase of walking and jogging and in single-leg squatting, pelvis and thorax obliquities were similar in ACLR, ACLD and control participants. Our hypothesis that individuals with ACL injury would have greater range of pelvis and thorax obliquities than controls was therefore not supported. The lack of group differences may be incorrectly interpreted as indicating that the ACL injured participants had recovered their movement patterns during these functional tasks; however, previous analyses of these same participants have revealed deficits in kinematics, motor control, and performance in the sagittal plane [13], [14], [15]. The current data therefore extend previous analyses and show that ACL injured individuals in the general population who have underdone physiotherapy show no residual deficits in pelvis and thorax movement in the frontal plane during the stance phase of walking and jogging and in single-leg squatting.

One quarter of the ACLD participants in this study felt unable to perform the single-leg hop for distance, and did not complete these trials. The single-leg hop for distance was the most challenging of the activities performed [14]. We speculate that it posed a threat to the stability of the knee joint that was particularly salient in ACLD knees, and that this resulted in a level of anxiety that caused one-quarter of ACLD participants to abstain. In support of this, the participants who felt unable to perform the single-leg hop for distance had a greater fear of movement than their counterparts who did perform the activity, indicated by greater TSK score.

Most existing studies of single-leg hop in ACLD individuals do not report any individuals who were unable or refused to perform a single-leg hop (e.g. [25], [26], [27], [28], [29], [30], [31], [32], [33], [34], [35], [36]). We are aware of only one existing report of ACLD individuals unable or unwilling to perform a single-leg hop [37], in which only four of 10 participants with poor dynamic knee stability performed the hop. The authors did not measure fear of movement, but do state that refusal to hop was due to “fear of further damaging their knee” (p265; [37]). Further study on this subgroup of ACLD individuals is warranted to identify if the avoidance behaviour is a manifestation of physical or psychological characteristics. The latter is commonly studied in chronic low back pain, where it is postulated to perpetuate chronic pain (cf. fear-avoidance model; e.g., [38]). Several studies have reported the relations between psychological characteristics and outcomes in ACLR individuals (see [39], [40]), but the extent to which psychological characteristics play a role in the behaviour of chronic ACLD individuals is less well studied.

The three-quarters of ACLD participants who did complete the single-leg hop for distance moved through a *smaller* range of pelvis and thorax obliquities than controls. This is contrary to our hypothesis. Although the results were significant, the magnitude of the difference was small and the clinical significance remains to be determined. In support of our results, Rudolph et al. [37] reported that the four ACLD participants with poor dynamic knee stability who did hop did so with a stiffer knee joint than controls. It is not possible to tell if this was a movement pattern learned during rehabilitation, an adaptation that allowed these individuals to feel confident in performing the movement, a response to feeling anxious about performing the movement, or a combination of these factors. Anxiety can increase the excitability of the motor system (e.g., [41]) and influence the control of static balance [42], [43], [44], [45] and gait [46]. However, the effect of fear or anxiety on the control of dynamic activities is not well studied. Interestingly, anxiety-induced changes that occur during static balance serve to increase the stiffness of the ankle joint [42], [43], [44], [45]. If similar changes occur globally across the body, this would fit well with our observation of reduced movement.

### 4.1 Implications for rehabilitation

In elite athletes, minimal movement of the pelvis and trunk in the frontal plane during dynamic single-leg activities is one marker of progression through rehabilitation after ACLR [11]. Our data show that individuals from the general population exhibited frontal plane movement of the pelvis and thorax during dynamic single-leg activities. The magnitude of this movement varied across individuals, but could be substantial, often close to 15° and reaching as high as 60°. Our data therefore show that the target of minimal frontal plane motion is not achieved by individuals in the general population without a history of ACL injury. Although our data indicate that minimal movement is not the ‘typical’ pattern observed in non-injured individuals in the general population, they cannot be used to address whether reducing frontal plane motion would affect injury risk. However, there is one note of caution to make on this topic. In the single-leg hop for distance, pelvis and thorax range of motion was smallest in ACLD participants. We have previously shown that this group exhibits deficits in lower-limb motor control in the sagittal plane in this task [13]; therefore, in these individuals, an over-reliance on frontal plane movement of the pelvis and thorax as an indicator of recovery may be misleading, and cause recovery to be overestimated.

The hypothesis that minimal trunk movement in the frontal plane during dynamic movements is optimal for reducing the risk of ACL re-injury is based on indirect evidence. The ability to resist trunk movement in the frontal plane after a sudden release of force was predictive of ACL injury [3], but it is not known how this generalises to trunk movement during dynamic activities. Lateral trunk lean away from the direction of a sidestep cutting task was positively associated with peak external knee abduction moment [4], [6], and knee abduction moment during a jump-landing task was a predictor of ACL rupture in female athletes [5]. Therefore, indirect evidence suggests that lateral trunk lean may influence ACL injury risk through an effect on external knee abduction moment. It remains to be determined if participants with a large range of frontal plane trunk or thorax movement are at higher risk of experiencing ACL injury.

### 4.2 Limitations

This was an analysis of existing data, and the study was not designed a priori to address frontal plane movements of the pelvis and thorax. The mechanism of injury (contact vs. non-contact) was not documented and it remains to be determined if mechanism of injury has an impact on the motion of the pelvis and thorax after injury. The inter- and intra-individual variability in thorax motion in the single-leg squat and hop activities may indicate that participants were still learning how to perform these activities, and it is not possible to state if a more stable pattern of movement would emerge with more practice. The data cannot be used to determine whether minimal frontal plane motion of the thorax and pelvis is optimal to prevent initial or re-injury of the ACL, and this remains an open question for future studies.

### 4.3 Conclusions

Minimal frontal plane motion was not the typical movement pattern observed in the general population, particularly in challenging dynamic activities. In these activities there was no consistent pattern of coordination between pelvis and thorax, and range of motion could be substantial. Consideration must be given to the possibility that, in this population, minimal movement may not indicate optimal performance when performing challenging tasks, but may indicate anxiety about performing the movement.
